# ESCRS guideline for cataract surgery 2024: executive summary

**DOI:** 10.1097/j.jcrs.0000000000002012

**Published:** 2026-08-26

**Authors:** Joukje C. Wanten, Victoria Till, Oliver Findl, Béatrice Cochener-Lamard, Thomas Kohnen, Rudy M.M.A. Nuijts

**Affiliations:** From the University Eye Clinic Maastricht, Maastricht University Medical Center, Maastricht, the Netherlands (Wanten, Nuijts); Vienna Institute for Research in Ocular Surgery (VIROS), A Karl Landsteiner Institute, Hanusch Hospital, Vienna, Austria (Till, Findl); Service d'Ophtalmologie, CHRU Brest, Brest, France (Cochener-Lamard); Université de Bretagne Occidentale, Brest, France (Cochener-Lamard); Department of Ophthalmology, Goethe University, Frankfurt, Germany (Kohnen).

## Abstract

Cataract is a leading cause of visual impairment globally, with surgery as the only effective treatment. Management involves a care pathway, including screening, patient selection, preoperative assessment, surgery, and postoperative care. The ESCRS developed evidence-based guidelines to aid decision-making for patients, clinicians, and stakeholders. A multidisciplinary panel, supported by methodologists using the Grading of Recommendations, Assessment, Development, and Evaluations approach, addressed 32 key clinical questions. Recommendations include intracameral antibiotics to prevent endophthalmitis, topical anesthesia as the preferred technique, and toric intraocular lenses for corneal astigmatism ≥1.0 diopter. Conventional and femtosecond laser-assisted surgeries are comparably safe and effective. Nonsteroidal anti-inflammatory drug eyedrops, alone or with corticosteroids, are recommended to reduce inflammation and prevent cystoid macular edema. Additional guidance includes shared decision-making for lens selection, safe use of immediate sequential bilateral cataract surgery, and potential of remote postoperative care to enhance healthcare efficiency. These guidelines aim to improve outcomes, patient experience, and care delivery.

## Abstract 2

This ESCRS cataract surgery guideline provides evidence-based recommendations, developed using the GRADE approach, to support decision-making, optimize outcomes, enhance patient experience, and promote efficient care delivery.

Cataract is a significant cause of blindness, which is currently only reversible by surgery. As age advances, the prevalence of cataracts increases significantly. The prevalence of cataracts ranges from 3.9% among individuals aged 55 to 64 years to as high as 92.6% among those aged 80 years and older. It is projected that by 2025, the worldwide population of individuals affected by cataract blindness will increase to 40 million.^1^ Cataract surgery is the most commonly performed surgical procedure worldwide, with 7 million cases each year in Europe, 3.7 million cases in the United States, and 20 million worldwide.^2^

The purpose of the ESCRS Cataract Surgery Guidelines is to address the value of diagnostic and therapeutic steps for various stakeholders in the patient cataract care pathway. The guideline aims to apply to all healthcare workers (eg, ophthalmologists, residents, general practitioners, nurses, optometrists, opticians, healthcare decision takers, patient societies, and healthcare insurance companies) and patients interested in cataract management. It provides explicit, evidence-based recommendations and insights that healthcare providers should follow to deliver high-quality care. The clinical recommendations are crucial for supporting clinical decision-making and promoting better care, transparency, and reduced unwanted practice variation. Although they are not prescriptive regulations, healthcare providers may deviate from these recommendations in complex cases where the patient's circumstances differ significantly from the “average patient.” The physician must exercise his/her judgment on the suitability of the care provided to a particular patient, considering all the circumstances presented by the patient. However, any deviation should be documented in the patient's record and be supported by clear reasoning. This documentation requirement is not intended to limit clinical autonomy but rather reflects standard medicolegal practice in many European countries and serves to document the shared decision-making process between physician and patient. These guidelines are developed within the European regulatory and medicolegal framework; therefore, the recommendations reflect current approvals and standards of care applicable in Europe.

This study represents a condensed summary of the ESCRS Cataract Surgery Guidelines and therefore includes only selected recommendations. Readers are strongly encouraged to consult the comprehensive version of the guidelines in Supplemental File 1 (available at http://links.lww.com/JRS/B687) for a detailed discussion of the evidence base, methodology, and full set of recommendations and considerations.

## METHODOLOGY

This guideline was developed according to the comprehensive quality criteria as described in the Appraisal of Guidelines for Research and Evaluation II instrument.^3^ The guideline development group consisted of 18 ophthalmologists, and the constituting guideline development group of 2 PhD candidates and a supervising methodologist. All clinical members possessed expertise in diagnosing and treating cataracts, and they were geographically distributed to cater to diverse regions. Review questions were formulated according to the PICO framework. Outcome parameters were selected based on importance for decision-making in the clinical setting. Literature searches were performed, using KSR evidence, CDSR, Medline, Embase, and Central as resources to identify relevant systematic reviews and randomized controlled trials for each review question, in total 5744 articles were found. The 2 PhD candidates performed selection of the articles supervised by the methodologist. Critical appraisal of available systematic reviews was performed based on the Risk of Bias assessment Tool ROBIS by reviewers at KSR Ltd.^4^ The quality of the relevant evidence was summarized using the Grading of Recommendations, Assessment, Development, and Evaluations (GRADE) approach.^5^ According to the GRADE approach, the evidence is classified as high (++++), moderate (+++), low (++), or very low (+). These classifications are accompanied by a specific formulation of the recommendations, using the wording “must,” “should,” “could,” “may,” “may not,” and “can be considered.” Considering high-level evidence, the term “must” was used in the recommendations of this guideline. In the case of moderate evidence, “should” or “could” were used. For low-graded evidence, “could” or “may” are applicable, and finally, when there was very low evidence implemented in the recommendations, “can be considered” was used.^4–6^

**Figure 1.**
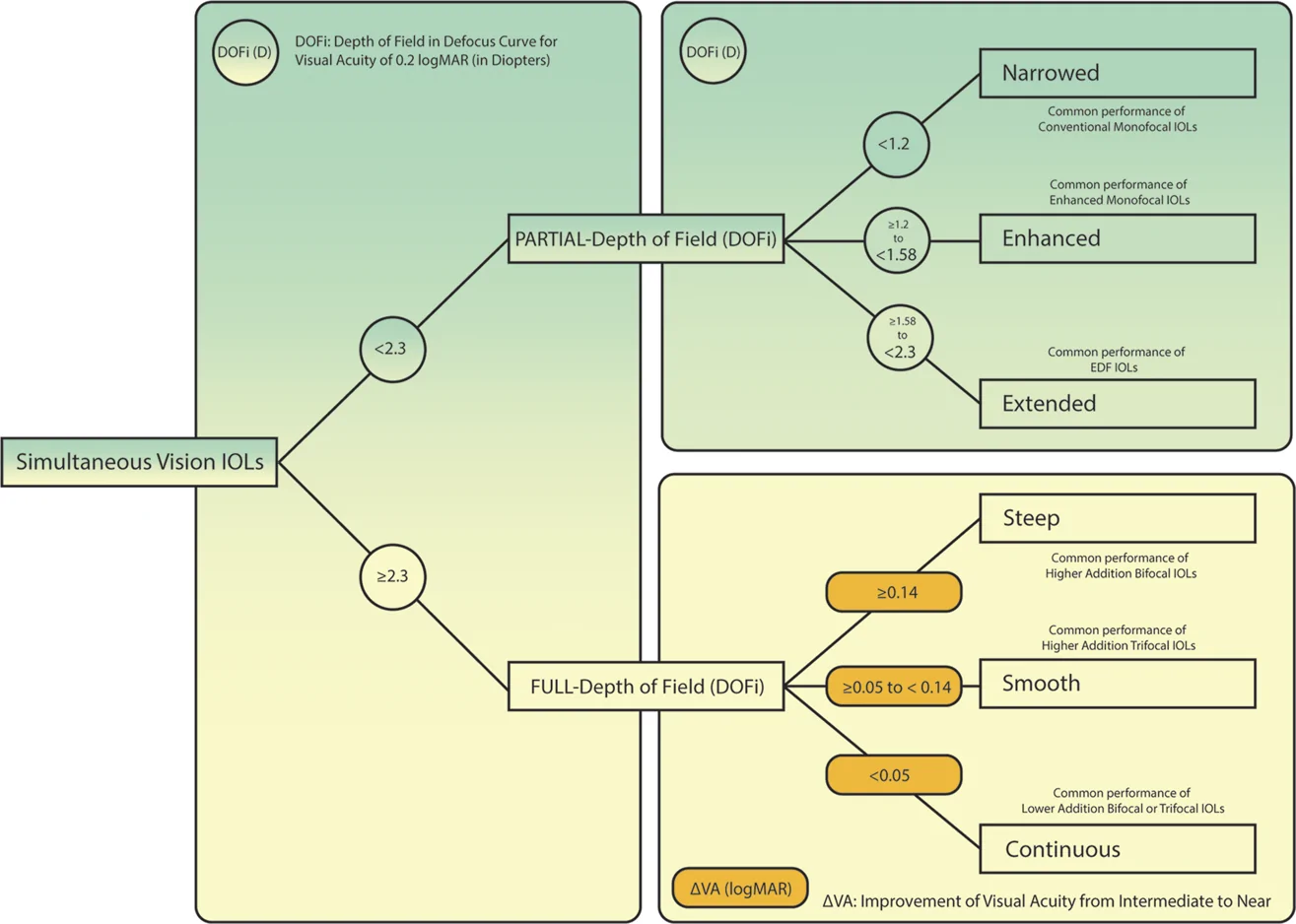
Diagram of functional classification depending on (1) the DOFi achieved in the monocular defocus curve with best correction at distance at the 0.20 logMAR VA level and (2) the improvement of VA from intermediate to near (ΔVA). Adapted from Ribeiro et al., J Cataract Refract Surg. 2024;50(8):794–798. DOFi = depth of field; ΔVA = VA increase from intermediate to near

## SUMMARY OF RECOMMENDATIONS

The structure of this guideline is according to the patient cataract care pathway and includes following sections: screening and patient selection, preoperative assessment, perioperative procedure, postoperative care, and complications.

### Screening and Patient Selection

#### What are the indications for cataract surgery? (Question 4.1)

A cataract may be initially suspected or identified by optometrists or other eyecare providers, but the formal clinical diagnosis is made by an ophthalmologist at the slitlamp examination. The decision to proceed with cataract surgery should be made jointly by the patient and ophthalmologist and documented in the patient's medical record (GRADE +).

Important aspects to be considered for the indication for cataract surgery are the presence and appearance of cataract, a patient's visual acuity and function (visual acuity and quality of vision), the subjective disability of the patient, and the expected benefits of the cataract surgery (GRADE +).

Comorbidities and the surgical risk profile should be considered and discussed with the patient before the surgery. Documentation of this process in the patient file is mandatory The use of validated patient satisfaction questionnaires and patient-reported outcome measures may be considered to evaluate the outcomes of cataract surgery and support quality improvement or benchmarking, but their use can be adapted according to local practice and regulatory requirements (GRADE +).

#### Will the presence vs absence of (characteristic A) impact efficacy and safety outcomes in patients for whom cataract surgery is considered? (Question 4.2)

The presence of patient characteristics and comorbidities can have an impact not only on the outcome in vision but also on the risks of surgery and postoperative complications. These should be discussed with the patient so that they are fully informed, and expectations are realistic (GRADE +).

Significant ocular comorbidities, such as diabetic retinopathy, glaucoma, maculopathies, uveitis, and strabismus, should be identified during the preoperative evaluation because its presence can affect the postoperative visual acuity and function (GRADE +).

In addition, certain risk factors can lead to a higher complication rate during cataract surgery including pseudoexfoliation syndrome, Fuchs endothelial dystrophy, shallow anterior chambers, white and brunescent cataracts, small pupils, and eyes with an extreme axial length (<22 mm or >26 mm). In patients who previously underwent refractive surgery, specific precautions should be taken to prevent a refractive surprise (see Question: Which formula(e) for calculating the intraocular lens in patients who have undergone refractive surgery is/are preferred?).

Regarding eyes with “active disease” (such as uveitis, proliferative diabetic retinopathy, neovascular age-related macular degeneration (AMD), and herpetic keratitis), these may be more susceptible to complications when performing cataract surgery.

It is recommended to perform the surgery when the disease is in a quiet phase. Customizing the postoperative follow-up based on the presence of ocular comorbidities is essential for ensuring comprehensive and effective care. Furthermore, it is crucial to provide patients with thorough information about potential risks to ensure realistic expectations (GRADE +).

Table 1 presents recommendations for patient characteristics and comorbidities associated with increased risk of surgical complications or suboptimal visual outcomes.

**Table 1.** Patient characteristics and comorbidities with an increased risk for complications during or after cataract surgery, and a suboptimal visual outcome

Comorbidity	Recommendation
Macular degeneration	Cataract surgery in patients with macular degeneration improves visual function in all severity grades of AMD, at least in the short-term (GRADE +) It is recommended to perform cataract surgery in a period of quiescence of neovascular AMD, but timing for cataract surgery must be individualized according to the patient's needs (GRADE +) Maculopathies should be identified before cataract surgery. The long-term effects of AMD after cataract surgery are still unclear (GRADE +)
Glaucoma/ocular hypertension	IOP should be monitored short-term after cataract surgery in patients with glaucoma (GRADE ++)
Diabetic retinopathy	The presence of active diabetic retinopathy or diabetic macular edema increases the risks of intraoperative and postoperative complications, such as macular edema (GRADE +) Cataract surgery can be considered when the underlying retinal disease is stabilized or when the presence of the cataract impedes with the evaluation and treatment of the retinal disease (GRADE +)
DED	Preoperative dry eye has an impact not only on preoperative examinations but also on postoperative outcomes. DED management should be optimized before surgery (see preoperative examinations), and the patients should be informed that DED symptoms often become worse after surgery (although often temporary) (GRADE +)
Fuchs endothelial corneal dystrophy	The severity of Fuchs endothelial dystrophy should be evaluated for cataract surgery decision-making, based on the clinical presentation and the visual symptoms (GRADE +)
Amblyopia	Preoperative orthoptic examination may be necessary to assess binocular vision and detect possible amblyopia (GRADE +)
Corneal opacities	Patients should be advised on the impact of the opacities on outcomes and the risk of additional medical or surgical management (GRADE +)
Macular pucker/ERM	Compared with eyes without ERM, higher rates of cystoid macular edema and a reduced postoperative gain in visual acuity can be noted (GRADE +)
Previous refractive surgery	In patients with cataract who previously underwent refractive surgery, special preoperative examinations such as corneal topography and tomography may be of added value (GRADE +) The impact of previous surgery on refractive outcome prediction should be discussed and the need for need for spectacles after surgery (GRADE +)
Shallow anterior chamber	Patients with a shallow anterior chamber should be informed about the increased risk of perioperative and postoperative complications such as iris prolapse and corneal endothelial cell loss (GRADE +)
PEX	PEX is an important risk factor in phacoemulsification because of complications such as poor pupillary dilation, zonular weakness inducing intraoperative or postoperative lens dislocation, vitreous loss, postoperative IOP spikes, late zonulopathy, capsular phimosis, prolonged inflammation, and postoperative corneal decompensation. Patients should be counselled accordingly (GRADE +)
White/Brunescent cataract	Surgical adaptions such as using trypan blue for capsular bag staining and decompression/aspiration, or “milking” of the cortical material should be performed to reduce the risk of capsular tear (GRADE +) High cohesive viscoelastics and intravenous mannitol may be used to reduce the risk of tear out (GRADE +) Anterior segment OCT may be performed to grade lens intumescence (GRADE +)
Small pupil/IFIS	The use of pupil expansion strategies should be considered in cases with small pupils that cannot be dilated pharmacologically (GRADE +) A staged approach of viscodilation, pupil expansion devices, including rings and iris hooks, should be considered, and these devices should be available in the operating room (GRADE +) In cases of IFIS, a combination of strategies including appropriate phacoemulsification fluidic parameters, pharmacological agents, longer corneal tunnels, and dispersive viscoelastics should be considered (GRADE +)
Eyes with extreme axial length	Long eyes can be defined as eyes with an axial length of more than 26 mm, while short eyes are generally defined as eyes with an axial length of less than 22 mm. Patients should be informed about the increased risk of refractive surprise and complications with postoperative refraction being noncoherent with target refraction (GRADE +)
Uveitis	Patients with uveitis will have visual improvement after cataract surgery but are also at more risk for the development of macular edema and a recurrence of uveitis (GRADE +) Active inflammation should be controlled before surgery, which means that the inflammation is sufficiently controlled, where possible (GRADE +) Preexisting corneal, anterior, and posterior segment pathological changes such as corneal scarring, band keratopathy, iris atrophy, vascular fragility, anterior and posterior synechiae, pupillary and cyclic membrane formation, macular scarring, optic nerve inflammation, ischemia, atrophy, and retinal vascular disease should be considered (GRADE +)
History of herpes keratitis	Antiviral prophylaxis with acyclovir or valacyclovir should be considered (GRADE +)
Vascular occlusions	Patients with preoperative history of central retinal vein occlusions should be informed about the possible limitations on their visual outcomes after cataract surgery compared with other patients without retinal diseases (GRADE +)

Abbreviations: AMD = age-related macular degeneration; DED = dry eye disease; ERM = epiretinal membrane, GRADE = Grading of Recommendations, Assessment, Development, and Evaluations; IFIS = intraoperative floppy-iris syndrome; PEX = pseudoexfoliation syndrome

#### What mental health factors must be considered when preparing for cataract surgery? (Question 4.3)

Cataract surgery has a beneficial effect on cognitive and mental health. Cataract extraction can be considered in patients at higher risk for cognitive decline and impaired vision due to cataracts (GRADE +/++).

The timing of the surgery regarding the course of the mental illness and the potential application of immediate sequential bilateral cataract surgery (ISBCS) may be of benefit for these patients (GRADE +/++).

#### What information about surgery, target refraction, and complications should be given to the patient before cataract surgery? (Question 4.4)

The ophthalmic surgeon should ensure that the following information is verbally provided to the patient before obtaining informed consent and performing cataract surgery:

- The option of not undergoing surgery
- Purpose and nature of the cataract surgery
- Surgery process overview
- Risk for (serious) complications during and after the surgery
- Patient-specific additional risks
- Surgery on 1 or both eyes
- Target refraction and expected vision improvement after surgery
- Treatment options: intraocular lens (IOL) types, including discussion of the potential need for spectacles after surgery, regardless of IOL choice The financial implications of the surgical and IOL choices
- In cases of bilateral cataract surgery: delayed sequential or immediate bilateral surgery
- Type of anesthesia
- A clear means to reach the surgeon or delegated assistant in case of emergencies

Targeted interventions improve patients' satisfaction with cataract surgery and the accompanying postoperative care (GRADE +).

In addition to verbal information, patients undergoing cataract surgery should be provided with written information and, if possible, audio-visual material. It is important to consider national informed consent guidelines and adapt the information provided to local best practices and legal frameworks (GRADE +).

#### In patients needing cataract surgery, what are the effects of immediate bilateral surgery compared with delayed sequential surgery and what is the minimum time between cataract surgery on the first and second eyes? (Question 4.5)

ISBCS is effective and safe, has a high degree of patient satisfaction, and can be considered in patients without complication-inducing ocular comorbidities (GRADE +).

There are comparable clinical outcomes of delayed sequential bilateral cataract surgery (DSBCS) and ISBCS. Therefore, either technique can be considered (GRADE +/+++ [for endophthalmitis]).

Bilateral cataract surgery on the same day allows rapid patient rehabilitation and helps avoid suboptimal visual function while waiting for second-eye surgery. However, there was no extra long-term benefit of self-assessed visual function compared with cataract surgery in 1 eye at a time (GRADE +).

Specific relative contraindications must be considered if bilateral simultaneous cataract surgery is planned:

- The ISBCS should be reconsidered if there is an increased risk of perioperative or postoperative complications.
- Second-eye surgery should generally be deferred in the event of major intraoperative complications in the first eye. Minor complications may allow second-eye surgery if judged safe by the surgeon.

If bilateral simultaneous cataract surgery is planned, it should be considered and treated as 2 entirely separate procedures, according to the principal practice guideline for bilateral surgery. These guidelines can be found at https://www.rcophth.ac.uk/wp-content/uploads/2020/09/Immediate-Sequential-Bilateral-Cataract-Surgery-Guidance.pdf.^7^

#### Do pseudophakic presbyopia-correcting IOLs have a better postoperative outcome than monofocal IOLs with a monovision target? (Question 4.6)

Detailed patient information must be given to choose the correct IOL types for patients undergoing cataract surgery with pseudophakic correcting presbyopia IOLs (GRADE +).

A comparison of visual outcomes, spectacle dependence, dysphotopsia risk, and associated costs for multifocal IOLs; extended depth of focus (EDF) IOLs; and monovision is provided in Table 2.

**Table 2.** Overview of visual performance, spectacle dependence, dysphotopsia risk, and cost for multifocal IOLs, EDF IOLs, and monovision

Multifocal IOLs	EDF	Monovision
Good near, intermediate, and distance visual acuity	Good intermediate and distance visual acuity	Good intermediate and distance visual acuity
Spectacle independence	Spectacles for near vision needed in most cases	Spectacles for near vision needed in most cases
Higher probability of dysphotopsia, reduced contrast sensitivity	Reduced probability of dysphotopsia	Low probability of dysphotopsia
Increased costs (depending on IOL/clinic)	Increased costs (depending on IOL/clinic)	Mostly covered by insurance

Multifocal IOLs should be considered in patients who desire a high chance of spectacle independence for far, near, and intermediate vision because multifocal IOLs show better results than standard monofocal IOLs in uncorrected near and intermediate vision (low certainty evidence for bifocal vs trifocal).

Thoughtful use of multifocal IOLs is recommended because unwanted visual phenomena such as halos, glare, and starbursts are more common with multifocal IOLs than with monofocal IOLs (GRADE ++).

EDF IOLs or pseudophakic monovision can be considered for patients seeking improved intermediate vision. Some designs of EDF IOLs may be associated with less dysphotopsia than multifocal IOLs, although this is not consistent across all designs (GRADE +).

#### Do toric IOLs give a better postoperative outcome than non-toric IOLs in cataract surgery? From which magnitude of corneal astigmatism is a toric IOL indicated? (Question 4.7)

Current recommendations are based on studies performed using anterior keratometry:

In the case of regular corneal astigmatism, toric IOLs may be considered for implementation (GRADE ++).

Toric IOLs should be considered in eyes with a degree of corneal astigmatism of 1.0 diopter (D) or more. The greatest clinical effectiveness is observed in eyes with corneal astigmatism >2.0 D, with meaningful visual benefits also observed from 1.5 D and above. Although toric IOLs may offer benefits in eyes with astigmatism ≥1.0 D, the supporting evidence is more limited (GRADE ++ for ≥2.0 D; GRADE + for ≥1.5 D).

New insights rely on predictions of postoperative astigmatism, making it imperative to use these predictions as a basis for decision-making in cases with corneal astigmatism.

#### What type of anesthesia is indicated for the patient? (Question 4.8)

There are several accepted and safe anesthesia techniques available for patients who undergo cataract surgery. Topical anesthesia seems to be the most commonly used anesthesia technique during cataract surgery (GRADE ++/+++).

For further reducing pain during the cataract surgery, additional intracameral lidocaine can be instilled (GRADE ++/+++).

The choice for a specific type of anesthesia during cataract surgery should be made together with the surgeon and patient (GRADE +).

Nevertheless, other techniques have also gained acceptance and have demonstrated safety during cataract surgery. The decision-making process regarding the choice of anesthesia should be a collaborative effort involving the ophthalmic surgeon and the patient. The anesthesia technique may vary from surgeon to surgeon, influenced by their experience and individual preferences. In addition, patient-specific considerations, such as medical history, anxiety levels, and comfort, are vital in determining the most suitable anesthesia approach.

Under certain conditions, preoperative fasting and intravenous sedation can be considered. When general anesthesia is needed, preoperative health tests and blood samples might be necessary.

### Preoperative Assessment

#### What kind of diagnostics and preoperative assessment of the patient should be performed? In patients who will undergo cataract surgery, what are the effects of diagnostic A vs no diagnostic A or vs diagnostic B on efficacy and safety outcomes? (Question 5.1)

Before cataract surgery, a series of essential preoperative examinations must be conducted to ensure a comprehensive understanding of the patient's ocular health and overall medical condition.

In general, for preoperative assessment before cataract surgery, the following diagnostic measures are recommended: refraction, visual acuity, slitlamp assessment, dilated fundus examination, optical biometry, and tonometry (GRADE ++).

In the presence of refractive astigmatism, additional measurements with tomography/topography are recommended (GRADE +).

It is recommended to provide the patient with detailed information (see Question: What information about surgery, target refraction and complications should be given to the patient before cataract surgery?) and use cataract-specific checklists adapted to the clinic of practice because checklist use is associated with improvement of patient safety by reducing surgical morbidity and mortality (GRADE +).

In addition to diagnostic measurements, providing detailed patient information is crucial. This includes discussing target refraction, potential visual outcomes, the possibility of needing spectacles after surgery, and postoperative instructions with the patient. Equally essential is providing a detailed explanation of the surgical process and potential complications.

#### What kind of diagnostics and preoperative assessments of patients who previously underwent refractive surgery should be performed? (Question 5.2)

It is recommended to perform corneal tomography or topography in patients who previously underwent corneal refractive surgery (GRADE +).

In post-radial keratotomy eyes, a corneal tomography/topography should be performed for assessment of corneal astigmatism and corneal irregularities (GRADE +).

A slightly myopic target refraction should be considered (GRADE +). Patients should be informed about the possibility of a refractive surprise and that spectacles may still be required for certain tasks after surgery (GRADE +). It is recommended to choose a slightly myopic target refraction in eyes with anterior and posterior phakic IOLs (GRADE +). An anterior segment optical coherence tomography (OCT) may be used for anterior chamber depth (ACD) measurements in anterior phakic IOLs (GRADE +). Endothelial cell count should be performed in patients with anterior phakic IOLs before cataract surgery (GRADE +).

#### In which patients with an indication for cataract surgery is posterior segment OCT indicated? (Question 5.3)

In general, posterior segment OCT in cataract surgery should be used when there is a clinical indication, such as AMD, diabetic retinopathy, glaucoma, uveitis, an epiretinal membrane, or when the visual acuity is worse than expected (GRADE +).

OCT is more effective in detecting optic nerve or macular pathologies than a regular fundus examination (GRADE +).

Posterior segment OCT may be used in routine cataract cases and can be considered at least in the following situations: (GRADE +)

- In case of increased risk or medical history of macular abnormalities that could adversely affect the postoperative visual outcome, such as AMD, diabetic retinopathy, glaucoma, uveitis, or an epiretinal membrane
- Where the visual acuity is worse than expected and cannot be fully explained by the degree of cataract
- In case of considering presbyopia correction IOLs

#### In which patients with an indication for cataract surgery is ultrasonography (A- or B-scan) indicated? (Question 5.4)

Ultrasound biometry (A- and/or B-scan) should be used when there is low visibility of the posterior segment, such as in mature and dense cataracts when optical biometry is not applicable or feasible (GRADE +). An A-scan provides valuable information about the axial length, while B-scan offers imaging of the posterior segment, enabling the detection of some underlying ocular conditions that may affect surgical planning and outcomes.

#### What are the indications for specific assessment examinations for patients with corneal comorbidities? (Question 5.5)

Preexisting corneal comorbidities, including dry eye disease (DED), Fuchs endothelial dystrophy, and corneal scars and opacities are crucial to identify before performing cataract surgery because this may influence the outcomes.

DED management should be optimized before surgery, and patients should be informed that symptoms of DED may worsen temporarily after surgery. Patients with preexisting DED should be recognized and diagnosed before cataract surgery by using questionnaires, testing the tear breakup time, corneal fluorescein staining, or Schirmer test (GRADE +).

The severity of Fuchs endothelial dystrophy should be evaluated for cataract surgery decision-making, based on the clinical presentation and the visual symptoms (GRADE +).

Identifying corneal opacities before cataract surgery is essential for estimating potential visual improvement. Ideally, corneal transplantation should be performed first to allow accurate biometry and optimal visual outcomes after healing. Cataract surgery may be considered before keratoplasty in selected cases—for example, when the cataract is dense and visual impairment from the cornea is uncertain, or when corneal transplantation is delayed because of donor availability or medical factors. Each case must be evaluated individually, with careful assessment of the benefits, risks, and timing of interventions for both cataract and corneal disease (GRADE +).

#### What are the indications for specific assessment examinations for patients with keratoconus? (Question 5.6)

In patients with keratoconus, stabilizing procedures before cataract surgery should be considered if the patient is at risk of progression (GRADE +).

When evaluating astigmatism in this patient population, the anterior, posterior, and total corneal astigmatism should be assessed to perform the most accurate IOL power calculations (GRADE +).

#### What preoperative assessment is necessary for presbyopia-correcting IOLs? (Question 5.7)

Patient selection for pseudophakic presbyopia-correcting IOLs should be based on the desire for spectacle independence and realistic patient expectations. Patients with additional ocular pathologies may not be ideal candidates for pseudophakic presbyopia-correcting IOLs (GRADE +).

For the preoperative assessment, before implantation of a presbyopia-correcting IOL additional assessments can be considered including evaluation of dry eye symptoms, stereopsis assessment, corneal topography/tomography, posterior segment OCT, and pupillometry (GRADE +).

#### What preoperative assessment is necessary for toric IOLs? (Question 5.8)

In the case of implantation of a toric IOL, the preoperative assessment should encompass not only general mandatory evaluations but also corneal topography and/or tomography (GRADE +). Methods which include measurements of factors such as the additional posterior corneal astigmatism and effective lens position are preferred for toric IOL calculation (GRADE +).

### IOL Power Calculation

In general, new formulas appear annually, and therefore, it may take some time before evidence-based value of these formulas has been established.

#### Which formula(s) for calculating lens power should be considered? (Question 6.1)

There is a tendency toward improved outcomes with newer-generation formulas because they show less trend error, meaning that they seem more consistent along the range of axial lengths. Traditional formulas can still be considered an acceptable option where newer formulas are not available (GRADE +).

The ESCRS IOL calculator can be considered for comparing different formulas. Find the corresponding link at https://iolcalculator.escrs.org.

#### Which formula(s) for calculating lens power in specific conditions should be considered? (Question 6.2)

Specific IOL formulas are recommended for eyes with certain conditions to ensure accurate outcomes. In extreme long and short eyes, new-generation formulas are recommended (GRADE +).

In eyes with keratoconus, all formulas tend to overestimate the IOL power, which may result in a hyperopic refractive outcome if not carefully planned. It is recommended to avoid traditional formulas other than SRK/T and to use keratoconus-specific formulas for more accurate outcomes. The Barrett true-K and Kane formulas for keratoconus seem to provide more accurate results, especially in more advanced stages. A slightly myopic target refraction is generally preferred over risking an unintended hyperopic refractive outcome (GRADE +). In patients with steep (>46 D) or flat corneas (<42 D), the Barrett Universal II (total keratometry) and EVO (total keratometry) formulas may be considered (GRADE +).

The Haigis formula should be considered for patients with an ACD >3.5 mm, while the Hoffer Q formula is suggested for a shallow anterior chamber (ACD <2.5 mm) (GRADE +).

#### Which formula(s) for calculating the IOL in patients who have undergone refractive surgery is/are preferred? (Question 6.3)

When performing IOL calculations in patients who have undergone refractive surgery, designated formulas and methods should be used such as those in the ASCRS postrefractive surgery calculator.

The ESCRS calculator is available at https://iolcalculator.escrs.org/.

#### Which target refraction is preferred in patients who will undergo cataract surgery? (Question 6.4)

The selection of a specific target refraction highly depends on the selected IOL, expectations, and preferences of the patient. The patient and ophthalmologist should take the shared decision for IOL target selection (GRADE ++).

### Perioperative Procedure

#### What are the differences between femtosecond-assisted laser cataract surgery (FLACS) and conventional phacoemulsification cataract surgery? (Question 7.1)

Both conventional cataract surgery and FLACS can be used as:

- They are both safe and effective procedures (GRADE +/++).
- Visual acuity and refractive outcomes are comparable (GRADE +/++).
- Overall intraoperative and postoperative complication rates are low and seem similar for both conventional phacoemulsification and femtosecond laser-assisted cataract surgery (GRADE +/++).

FLACS may be considered in patients with dense cataract or low endothelial cell counts reflecting a short-term benefit without evidence of sustained long-term superiority (GRADE +/++).

#### What is the role of femtosecond laser in astigmatism control during cataract surgery? (Question 7.2)

FLACS and manual corneal incisions (eg, opposite clear corneal incisions, limbal relaxing incisions, and astigmatic keratotomies) are safe and effective options for astigmatism control during cataract surgery (GRADE +).

Femtosecond lasers can be used to perform corneal incisions specifically designed to correct corneal astigmatism (eg, intrastromal and penetrating femtosecond laser astigmatism keratotomies). These are more precisely performed than when done by hand (GRADE +).

#### What are the differences between different marking techniques for patients receiving toric IOLs? (Question 7.3)

Digital marking may result in less axis misalignment, a smaller difference vector, and less postoperative astigmatism than manual marking, but there are no clinically significant differences in visual and refractive outcomes between the 2 techniques (GRADE +).

#### What prophylaxis should be administered during cataract surgery to minimize the risk of postoperative endophthalmitis? (Question 7.4)

Intracameral antibiotic therapy should be used because it is effective and safe for preventing endophthalmitis after cataract surgery. The use of intracameral antibiotics significantly reduces the risk of endophthalmitis (GRADE +).

An intracameral injection should be used (cefuroxime 1 mg in 0.1 mL) at the end of the cataract surgery to lower the risk for postoperative endophthalmitis (GRADE +++).

Adequate antisepsis can be achieved by applying povidone–iodine 5% to 10% 3 minutes before commencing cataract surgery on the periocular skin, while lower concentrations (0.3% to 0.5%) are used for application to the conjunctival fornix. In cases of povidone–iodine allergy, chlorhexidine (0.02%) can be used as an alternative (GRADE +).

These recommendations reflect the current European regulatory framework; intracameral antibiotics approved and commonly used in other regions may demonstrate acceptable safety and efficacy but are not addressed as formal recommendations within these guidelines.

#### What prophylaxis should be used in cataract surgery to minimize the risk of postoperative inflammation? (Question 7.5)

- What is the most effective treatment to reduce postoperative inflammation after cataract surgery and reduce the risk of cystoid macular edema (CME)?
- Is perioperative inflammatory prophylaxis (dropless cataract surgery) equally effective as a postoperative anti-inflammatory eye drop regimen?

For the prevention of CME after routine cataract surgery in nondiabetic patients, topical nonsteroidal anti-inflammatory drug (NSAID) eyedrops or combination therapy with a corticosteroid can be used because both have similar clinical effectiveness in uneventful cases (GRADE +/++). Economic considerations may favor combination therapy in certain settings. Potential adverse effects of topical NSAIDs, including their use in patients with severe DED or collagen vascular disorders, have to be considered (GRADE +).

It is currently unclear whether dropless inflammatory prophylaxis is as safe and effective as topical inflammatory prophylaxis to prevent CME and inflammation after cataract surgery (GRADE +/++).

#### What are the optimal intraoperative and postoperative medication for patients with other ocular pathologies who undergo cataract surgery? (Question 7.6)

Specific postoperative treatments should be considered for patients with certain ocular comorbidities after cataract surgery.

In diabetic patients without diabetic retinopathy, it is recommended to use a combination of corticosteroid and NSAID eyedrops to prevent CME (GRADE +/++).

In patients with diabetic retinopathy, a supplementary subconjunctival depot of triamcinolone should be considered to reduce this risk. Intraocular pressure (IOP) must be monitored postoperatively when using a triamcinolone depot (GRADE +).

The literature reports discrepancies concerning the effect of antivascular endothelial growth factor (VEGF) intravitreal therapy in preventing CME after cataract surgery in patients with diabetes (GRADE +/++).

In patients with retinal diseases, topical NSAIDs should be used, and only in selected cases, intravitreal anti-VEGF injections could be considered (GRADE +).

In patients with uveitis, an increased frequency and prolonged topical steroid treatment is suggested. Oral steroids should be reserved for cases with severe or active intraocular inflammation, involvement of the posterior segment, or when topical therapy alone is insufficient to control inflammation preoperatively and postoperatively (GRADE +). Patients with glaucoma should receive carbonic anhydrase inhibitors or topical agents postoperatively to minimize the potential increase in IOP after surgery (GRADE +).

Oral acetazolamide administration postoperatively can be considered to reduce IOP elevation after cataract surgery (GRADE +).

Patients should be screened for risk factors for DED, particularly for meibomian gland dysfunction. Patients with DED should use artificial tears both before and after surgery to manage symptoms and optimize ocular surface health (GRADE +/++).

### Postoperative Care

#### What precautions does the patient have to consider after the surgery? When should the next follow-up visit take place? (Question 8.1)

The following precautions have to be considered after surgery: The patient should take the eyedrops as instructed and seek medical attention if vision decreases after prior improvement or if there is sudden onset of black dots, flashing lights, increased pain, or redness of the operated eye. Patients should not rub the eye and should not drive after surgery until cleared in line with national driving regulations (GRADE +).

New spectacles can be prescribed after 4 to 6 weeks, earlier refraction may be appropriate in selected patients once refractive stability is achieved. Uneventful cases can defer follow-up visits by up to 2 weeks without safety reduction (GRADE +).

#### What is the preferred postoperative medication that should be administered to treat inflammation and CME after cataract surgery? (Question 8.2)

This section addresses the management of CME once it has developed, rather than strategies for its prevention.

The incidence of clinically significant CME after cataract surgery has been reported to be as high as 2%. In many cases, the CME is a self-limiting condition that resolves spontaneously without causing any visual impairment.^8^ Nevertheless, there are instances where CME may persist or may lead to deterioration of the visual function, necessitating treatment. The primary treatment options for CME after cataract surgery are topical NSAIDs or steroids. Currently, there is insufficient evidence to determine an optimal treatment strategy for this condition. This highlights the importance of conducting future research to further explore and clarify the most effective strategies for managing CME after cataract surgery (GRADE ++).

No definitive conclusions can be drawn regarding the clinical effectiveness of injectable medications (including intravitreal injection of anti-VEGF, sub-Tenon steroid injections, and intravitreal steroid implants) for the treatment of CME (GRADE +).

#### When is remote care after cataract surgery indicated for patients? (Question 8.3)

Postoperative remote care after cataract surgery may serve as an alternative to short-term clinical examinations, potentially improving resource allocation and enhancing time and cost efficiency in surgical centers. However, the accuracy and validity of remote care and telemonitoring still require evaluation (GRADE +).

Screening has to be performed before allocating patients to a certain group that will receive remote care. Patients at an increased risk of complications or patients with comorbidities which may adversely affect their postoperative outcome should be prioritized for traditional postoperative hospital care (GRADE +).

### Complications

The complications included were therefore limited to the most common and clinically relevant events, based on available evidence and expert consensus. We acknowledge that less frequent or highly specific complications, while clinically important, are not comprehensively listed.

#### What kind of (serious) complications can occur during cataract surgery? (Questions 9.1 and 9.2)

Cataract surgery is generally regarded as a safe procedure with a low incidence of complications. However, intraoperative complications can occur and may vary in severity. Serious adverse events include anterior and posterior capsule rupture, dropped nucleus, zonular dialysis without vitreous loss, iris or IOL damage, and suprachoroidal hemorrhage. Other intraoperative complications may include anterior capsule tear and iris trauma.

#### What kind of (serious) adverse events can occur after cataract surgery? (Questions 9.3 and 9.4)

Postoperative complications may also vary in severity and timing. Serious adverse events include intraocular inflammation (such as CME), endophthalmitis, toxic anterior segment syndrome, retinal detachment, pseudophakic bullous keratopathy, retained lens fragments, and IOL-related issues such as dislocation, luxation, malposition, damage, opacification, calcification, residual refractive error, and photic phenomena. Other postoperative complications may involve posterior capsular opacification, capsular contraction syndrome, elevated IOP, persistent inflammation, corneal edema, binocular imbalance, diplopia, dry eye symptoms, and refractive surprise.

### Cost-Effectiveness

#### What is the cost-effectiveness of specific cataract surgery-related decisions? (Question 10)

A cost-effectiveness overview is provided in Chapter 10 (Supplemental File 1, available at http://links.lww.com/JRS/B687), including the following topics: endophthalmitis prevention, prevention of inflammation/CME after cataract surgery, toric IOLs, ISBCS, and femtosecond laser-assisted surgery. The main findings are summarized as follows:

Endophthalmitis prevention: Prophylactic intracameral cefuroxime significantly reduces the risk of endophthalmitis and is cost-effective compared with no antibiotic use. The incremental cost-effectiveness ratio (ICER) was €2427 per quality-adjusted life year (QALY).^9^

Inflammation and CME prevention: In nondiabetic patients, combination therapy with bromfenac and dexamethasone is cost-effective compared with monotherapy, with an ICER of €6544 per QALY.^10^ In diabetic patients, subconjunctival triamcinolone alone is more cost-effective than combination therapy with triamcinolone and intravitreal bevacizumab, with an ICER of €321 984 per QALY for the combination therapy.^11^

Toric IOLs: Bilateral toric IOL implantation in patients with corneal astigmatism was associated with slightly lower QALYs and was not considered cost-effective compared with monofocal IOLs, with reported ICERs ranging from €2500 to €20 000 per QALY.^12^

FLACS: FLACS does not offer clinical or economic advantage over conventional phacoemulsification, with reported ICERs showing higher costs and no added benefit (ICER: £167 120/QALY).^13^

ISBCS: ISBCS demonstrates superior cost-effectiveness compared with DSBCS, with ICERs ranging from €2500 to €80 000/QALY.^14^

Cost-effectiveness analyses are inherently dependent on country-specific pricing, reimbursement structures, and healthcare systems, and therefore relate to the specific interventions evaluated in the underlying studies. As a result, these findings cannot be generalized to entire drug classes or universally applied across different healthcare settings.

## LIMITATIONS AND IMPLEMENTATION CONSIDERATIONS

The aim of this guideline is to provide comprehensive and evidence-based recommendations for managing cataract surgery. However, it is important to acknowledge certain limitations. Primarily, there are persistent evidence gaps in specific areas, which may necessitate careful interpretation of certain recommendations. The ESCRS has supported various clinical studies, including the Endophthalmitis Study and PREMED Study, but further research projects are needed to address the knowledge gaps identified in this guideline. Moreover, it is crucial to recognize that the guideline primarily focuses on patients with routine cataract. Cases involving severe comorbidities may require individualized decision-making regarding outcomes or the surgery itself. In addition, the implementation of these recommendations may face barriers due to the variability in clinical practices and workflows. It is important to acknowledge these potential barriers because they may require tailored approaches in certain instances. Despite these limitations, this guideline serves as a valuable resource for all stakeholders involved in cataract care, providing evidence-informed guidance for cataract management.

## CONCLUSION

This guideline presents valuable insights and practical recommendations for healthcare professionals engaged in cataract care, achieved through a rigorous review of evidence and expert consensus. By adhering to these evidence-based guidelines, clinicians can elevate the quality of care they deliver to patients, thereby enhancing outcomes and overall health. We urge stakeholders to integrate this guideline into their clinical decision-making processes, recognizing its capacity to instigate positive changes in healthcare practice and patient outcomes. It is important to note that this document is dynamic and can be adapted as new evidence emerges. The ESCRS has played a crucial role in optimizing future cataract care by providing a transparent, evidence-based guideline, and supporting clinical studies in the field.

The extended version of the guidelines and all critical appraisals, and the comments overview can be found in Supplemental Files 1, 2, and 3 (available at http://links.lww.com/JRS/B687, http://links.lww.com/JRS/B688, and http://links.lww.com/JRS/B689).

## KEY RECOMMENDATIONS

The principal recommendations of this guideline are summarized below, structured by the corresponding level of evidence:

1. An intracameral injection should be used (eg, cefuroxime 1 mg in 0.1 mL) at the end of the cataract surgery to lower the risk for postoperative endophthalmiti (GRADE +++).
2. Topical anesthesia seems to be the most commonly used anesthesia technique during cataract surgery (GRADE ++/+++). For further reducing pain during the cataract surgery, additional intracameral lidocaine can be instilled (GRADE ++/+++).
3. Toric IOLs should be considered in eyes with a degree of corneal astigmatism of 1.0 D or more. The greatest clinical effectiveness is observed in eyes with corneal astigmatism >2.0 D, with meaningful visual benefits also observed from 1.5 D and above. Although toric IOLs may offer benefits in eyes with astigmatism ≥1.0 D, the supporting evidence is more limited (GRADE ++ for ≥2.0 D; GRADE + for ≥1.5 D).
4. The selection of a specific target refraction highly depends on the selected IOL, expectations, and preferences of the patient. The patient and ophthalmologist should take the shared decision for IOL target selection (GRADE ++).
5. The use of NSAIDs eyedrops, either in combination with corticosteroid eyedrops or as monotherapy is effective to use after routine cataract surgery to prevent inflammation and CME. However, there is a lack of sufficient evidence to establish the optimal treatment approach for this condition (GRADE +/++).
6. Both conventional cataract surgery and FLACS can be used because they are both safe and effective procedures (GRADE +/++). They give comparable visual acuity and refractive outcomes and overall intraoperative and postoperative complication rates (GRADE +/++).
7. In diabetic patients without diabetic retinopathy, it is recommended to use a combination of corticosteroid and NSAID eyedrops to prevent CME (GRADE +/++). In patients with diabetic retinopathy, a supplementary depot of triamcinolone should be considered to reduce this risk. IOP must be monitored postoperatively when using a triamcinolone depot (GRADE +).
8. ISBCS is effective and safe, has a high degree of patient satisfaction, and can be considered in patients without complication-inducing ocular comorbidities (GRADE +).
9. EDF IOLs or pseudophakic monovision can be considered for patients seeking improved intermediate vision. Some designs of EDF IOLs may be associated with less dysphotopsia than multifocal IOLs, although this is not consistent across all designs (GRADE +).
10. In general, posterior segment OCT in cataract surgery should be used when there is a clinical indication, such as AMD, diabetic retinopathy, glaucoma, uveitis, an epiretinal membrane, or when the visual acuity is worse than expected (GRADE +).
11. Patient selection for pseudophakic presbyopia-correcting IOLs should be based on the desire for spectacle independence, and realistic patient expectations. Patients with additional ocular pathologies may not be ideal candidates for pseudophakic presbyopia-correcting IOLs (GRADE +).
12. In the case of implantation of a toric IOL, the preoperative assessment should encompass not only general mandatory evaluations but also corneal topography and/or tomography (GRADE +). Methods which include measurements of factors such as the additional posterior corneal astigmatism and effective lens position are preferred for toric IOL calculation (GRADE +).
13. Specific IOL formulas are recommended for eyes with certain conditions to ensure accurate outcomes. In extreme long and short eyes, new-generation formulas are recommended (GRADE +).
14. Postoperative remote care after cataract surgery may serve as an alternative to short-term clinical examinations, potentially improving resource allocation and enhancing time and cost efficiency in surgical centers. However, the accuracy and validity of remote care and telemonitoring still require evaluation (GRADE +).

## PLANS FOR UPDATING THESE GUIDELINES

The ESCRS aims to update its guidelines periodically, every 5 years. This process will involve conducting update searches and assessing any relevant research in relation to the current recommendations and considerations. The project team will evaluate whether modifications or revisions to the recommendations are warranted in light of emerging evidence or evolving best practices.

## DEFINITIONS

### Definitions of Target Refraction

Before cataract surgery, patients should be consulted regarding the desired target refraction. This guideline covers the following target refraction goals:

- **Emmetropia**: Emmetropia refers to the condition in which there are no refractive errors present. When the eyes are in an emmetropic state, objects located at infinity are sharply focused on the retina without any need for accommodation. In practice, a refraction ranging between +0.25 D and −0.25 D is defined as emmetropia.^15^
- **Mini-monovision**: Mini-monovision refers to the condition where one eye (usually the dominant eye) is targeted for emmetropia, while the other eye (usually the nondominant eye) is targeted for slight myopia ranging between −0.25 D and −0.75 D to increase spectacle independence.^16^
- **Monovision**: Monovision refers to the condition when one eye is targeted for distance vision, while the other eye is targeted for near vision. The range of diopters for monovision correction may vary according to the specific needs of the patient and discretion of the surgeon. In practice, monovision ranges from −1.00 to −2.00 D.^17^

### Definitions of Different Types of Astigmatism

**Grading**
  - Low astigmatism (0.25 to 1.5 D)
  - Moderate astigmatism (1.5 to 3.0 D)
  - High astigmatism (above 3.0)
  - Myopic/hyperopic/mixed astigmatism.^18^
**Regular astigmatism**
  - With-the-rule astigmatism: Steep axis of the cylinder is vertical or within 30 degrees of the 90 degrees of vertical meridian (60 to 120 degrees).
  - Against-the-rule astigmatism: Steep axis of the cylinder is horizontal or within 30 degrees of the horizontal meridian (0 to 30 or 150 to 180 degrees).
  - Oblique astigmatism: Steep axis of the cylinder is not within 30 degrees of the horizontal or vertical meridians (31 to 59 degrees and 121 to 149 degrees).
**Irregular astigmatism**
  - Where the 2 main axes of astigmatism are not symmetric and/or do not lie 90 degrees apart (orthogonal).
  - Irregular or pathological astigmatism treatment is beyond the scope of these guidelines (eg, those caused by corneal dystrophies, trauma, degeneration, ocular surface disease, corneal ectatic diseases such as keratoconus, and prior corneal surgery).

### Definitions of Different IOLs

Classifying IOL technologies is not an easy task, primarily due to the various categories that can be integrated into a classification. Some of these categories represent different characteristics that may be inappropriately combined in an attempt to create a simplified taxonomy, which is not always feasible. Therefore, when defining an IOL, it is crucial to differentiate between various categories and avoid mixing them, to prevent confusion for the user.

#### Optical Technologies

Two main types of optical technologies, diffractive and refractive, have historically been used to classify IOLs, depending on the optical principles used for focusing light.^19^ Diffraction and refraction can be achieved through distinct optical structures or optical features.^20,21^ Diffraction can be accomplished with small optical apertures and diffractive gratings, while refraction can be achieved by varying the asphericity and radius of an optical surface or through zones and sectors.^19,21^ However, some designs may combine some of the previously described optical features or mechanisms; thus, controversies are likely to occur forcing to assign an IOL to 1 optical classification.^19,22,23^

According to the shape and number of foci, a taxonomy with 5 categories has been described in the literature: multifocal IOLs (including bifocal and trifocal), with the first trifocal lens commercially introduced in 2010; EDF, which emerged in 2014; monofocal IOLs with EDF (Mono-EDF) for which the first Conformité Européenne mark was granted in 2019; and conventional monofocal aspheric and finally spherical IOLs.^19,22,24–27^

#### Standard Terms and Definitions

According to the International Organization for Standardization (ISO 11979-7, 2024), there are 4 main categories of IOLs that are determined by optical design and/or clinical characteristics or performance^28^: monofocal, toric, simultaneous vision lens, and accommodating IOLs. From these, simultaneous vision lens are those nonaccommodative lenses that provide simultaneous vision at multiple distances and can be subclassified in 3 types:

- Multifocal: lens implants that emphasize optical and functionally useful acuity levels at far, but when compared with the monofocal control lens, also have improved optical and clinical performances at near focal distances.
- EDF: lens implants that emphasize optical and functionally useful acuity levels at far but also from far through intermediate focal distances.
- Full visual range: lens implants that emphasize optical and functionally useful acuity levels at far but also from far through intermediate and up to near focal distances.

#### Evidence-Based Functional Classification

A functional classification has been developed considering the end-points described in the standards, especially those referring to the depth of field measured through monofocal visual acuity defocus curves with the best distance correction.^29^ The functional classification can be found in Figure 1. This classification has been qualified as evidence-based because the scientific method (cluster analysis) has been the pillar during the development.

The methodology and metrics used for the cluster analysis are described in the Extended Version of the Guidelines (Supplemental File 1, available at http://links.lww.com/JRS/B687) in Chapter 2.4.

#### Conclusion

This IOL classification has been proposed by members of this ESCRS guideline development group. Historical terminologies used for classifying IOLs have been reviewed, and a functional classification has been introduced to enhance understanding, focusing on the visual acuity that patients achieve across the visual range. The importance of separating classification categories is emphasized because different optical technologies could produce similar functional outcomes.
